# Non-ST-Segment Elevation Myocardial Infarction As Initial Thrombotic Event of Thrombotic Thrombocytopenic Purpura: A Rare Challenging Case

**DOI:** 10.7759/cureus.36363

**Published:** 2023-03-19

**Authors:** Khalid H Mohamed, Saher T Shiza, Iqra Samreen, Adesola A Agboola, Alaa S Mohamed, Pavan Kumar Reddy Kalluru, Muhammad Haseeb, Rana Zohaib Munawar, Hira Nasir

**Affiliations:** 1 Neurology, Sheffield Teaching Hospitals NHS Foundation Trust, Sheffield, GBR; 2 Internal Medicine, Deccan College of Medical Sciences, Hyderabad, IND; 3 Pathology and Laboratory Medicine, Dele Hospitals, Lagos, NGA; 4 Neurology, Augusta University, Augusta, USA; 5 Obstetrics and Gynecology, Sri Venkateswara Medical College, Tirupati, IND; 6 Internal Medicine, Allama Iqbal Medical College, Lahore, PAK; 7 Internal Medicine, Mount Sinai Hospital, Brooklyn, USA; 8 Internal Medicine, Mayo Hospita, Lahore, PAK; 9 Internal Medicine, Mayo Hospital, Lahore, PAK

**Keywords:** thrombotic microangiopathies, acute coronary syndrome, myocardial infarction, thrombotic thrombocytopenic purpura, acquired ttp

## Abstract

Thrombotic thrombocytopenic purpura (TTP) is a rare autoimmune and devastating blood disorder that results in micro-clots throughout the body, leading to tissue damage and organ dysfunction resulting in widespread microangiopathic hemolytic anemia, thrombocytopenia, fever, and neurological symptoms. TTP patients commonly manifest renal and neurological symptoms; however, cardiovascular involvement is not widely reported in the literature. We report a case of non-ST-segment elevation myocardial infarction (NSTEMI) as an initial manifestation of TTP. Although rare, TTP complications must be considered among other possible causes of unexpected thrombocytopenia during acute phase treatment of acute coronary syndrome because of high morbidity and mortality.

## Introduction

Thrombotic thrombocytopenia-induced myocardial infarction (TTIMI) is a rare, potentially life-threatening condition that occurs as a result of thrombotic thrombocytopenic purpura (TTP) [1]. TTP is a rare autoimmune disorder resulting in micro-clots forming throughout the body. These micro-clots can obstruct blood vessels, leading to tissue damage and organ dysfunction resulting from widespread microangiopathic hemolytic anemia due to activation of the coagulation cascade [2]. TTP is caused by an autoimmune response, where the body's immune system attacks its own cells and tissues, forming micro-clots. The exact cause of TTP is believed to be related to a deficiency in the enzyme a disintegrin-like, and metalloproteinase with a thrombospondin type 1 repeat motif, member 13 (ADAMTS13), which is responsible for breaking down von Willebrand factor (vWF), a protein that helps blood clot. Without enough ADAMTS13, vWF can build up in the bloodstream, leading to the formation of micro-clots [3]. In TTP, the patient commonly manifests renal and neurological symptoms; however, cardiovascular involvement is not widely reported in the literature [4]. Herein, we present a case of myocardial infarction (MI) as an initial thrombotic event of TTP, masking other symptoms.

## Case presentation

**Figure 1:**
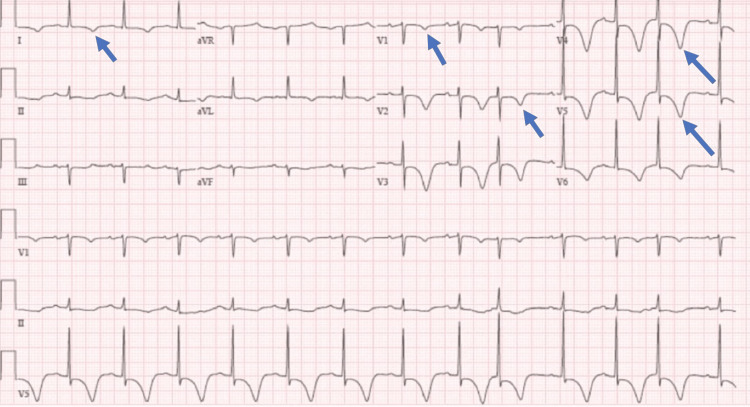
Electrocardiogram manifesting T-wave inversion in lead I and leads V1-V6.

A 45-year-old female with a history of hyperthyroidism and hypertension was brought to the emergency department with complaints of severe left sided-chest pain for the last three hours, which was worsening, retrosternal, radiating to left shoulder and neck, and with no aggravating and relieving factors associated with nausea, headache, and two episodes of watery projectile vomiting. She also reported having malaise and low-grade fever for the last two weeks. She complied with her medications and reported no history of polyuria, dysuria, or abdominal pain. On examination, she looked anxious, with a blood pressure of 140/90 mmHg, respiratory rate of 23/minute, and heart rate of 96/minute. An urgent electrocardiogram (EKG) revealed T-wave inversion in anterior, septal, and lateral leads (Figure 1). Initial laboratory results were unremarkable except for elevated serum cardiac troponin I level of 4.5 ng/dL (0-0.04), serum creatinine of 1.6 mg/dL, anemia, and thrombocytopenia.

She was diagnosed with non-ST segment elevation MI (NSTEMI) and managed with a loading dose of 300 mg aspirin, 300 mg clopidogrel, and morphine. She underwent transthoracic echocardiography, which revealed apical hypokinesia with an ejection fraction of 48%. She underwent coronary angiography showing minimal narrowing of the marginal branch and left main coronary artery and significant obstructive lesions in the proximal segment of the anterior descending coronary artery. Subsequently, she underwent percutaneous cutaneous intervention (PCI). She was commenced on metoprolol, enalapril, aspirin, atorvastatin, and clopidogrel. She reported clinical improvement and was discharged two days later.

Three days later, she presented again with fever, myalgia, diffuse arthralgia, and decreased urine out. She was vitally and hemodynamically stable. Repeat EKG was unremarkable except for old ischemic changes. Laboratory results were significant for anemia, thrombocytopenia, and deranged renal function test (Table 1). Antiplatelet therapy was discontinued, and her condition worsened with neurological deterioration. Further investigations revealed elevated lactate dehydrogenase, haptoglobin with normal prothrombin, partial thromboplastin time, and international normalized ratio. Peripheral smear revealed several schistocytes. ADAMTS-13 level in the blood was low, with elevated inhibitor titer and negative anti-nuclear antibody level.

**Table 1:** Results of the laboratory investigations. INR: international normalized ratio, ADAMTS: a disintegrin-like and metalloproteinase with thrombospondin motif.

Parameter	Lab value	Reference range
Hemoglobin	10.1 g/dL	12.5-15.5
Platelet count	31,000/µL	150,000-350,000
Red cell count	4.1 million cells/µL	4.7-6.1
Lactate dehydrogenase	998 IU/L	90-192
Prothrombin time	13 second	11-13.5
Haptoglobin	24 mg/dL	34-200
Partial thromboplastin time	27 second	25-35
INR	0.8	0.8-1.1
Serum creatinine	2.1 mg/dL	0.4-1.3
ADAMTS-13	19%	< 66%
ADAMTS-13 antibody titer	102 IU/mL	< 12

A provisional diagnosis of TTP was made, and she was managed with daily plasma exchange with fresh frozen plasma and prednisolone (1 mg/kg/day) followed by weekly rituximab. Her symptoms started improving with a remarkable increase in platelet count and improved urine output and consciousness level. She was maintained on clopidogrel, atorvastatin, and enalapril. She was maintained on steroids and rituximab for six weeks with regular follow-ups.

## Discussion

MI as an initial event of TTP is rare due to the formation of microthrombi in cardiac vessels. The exact prevalence of TTP-induced MI is unknown, and the literature underlines the prevalence of TTP-induced cardiovascular complications from 9.5% to 77%. However, TTP-induced MI as presenting event of TTP is extremely rare [5]. We have tabulated the reported cases of TTP-induced cardiac events in Table 2 [4-8].

**Table 2:** Reported cases of TTP-induced acute coronary syndrome. M: male, F: female, STEMI: ST-segment elevation myocardial infarction, NSTEMI: non-ST-segment elevation myocardial infarction, ADAMTS: a disintegrin-like and metalloproteinase with thrombospondin motif, TTP: thrombotic thrombocytopenic purpura.

Author et al.	Age/sex	Cardiovascular manifestation	Cardiovascular diagnosis	Platelet count	TTP manifestation	TTP diagnosis	Management
Gheith Z et al. [4]	68/F	Substernal chest pain	N-STEMI	30,000/ul	Fatigue, pallor, mental changes	Schistocytes, decreased ADAMTS-13	Plasmapheresis, steroids
Ghodsi S et al. [6]	29/M	Substernal chest pain, dyspnea	STEMI	17400/ul	Fever, oliguria, mental changes	Schistocytes, decreased ADAMTS-13	Plasmapheresis, steroids, cyclophosphamide
Takimoto et al. [7]	80/M	Chest pain	STEMI	10,000/ul	Malaise, oliguria	Schistocytes, decreased ADAMTS-13	Plasmapheresis, steroids
Dahal S et al. [8]	46/M	Left-sided chest pain	STEMI	13,000/ul	Fever, hematuria, altered sensorium	Schistocytes, decreased ADAMTS-13	Plasmapheresis, steroids
Salaru DL et al. [5]	49/M	Typical angina	STEMI	< 50,000/ul	Fever, anuria	Decreased ADAMTS-13	Steroids

Myocardial microthrombi in TTP results in MI and significantly contributes to high morbidity and mortality. The patient, in this case, had an NSTEMI that preceded the clinical diagnosis of TTP, which is different from those presented in previous studies. During the treatment of MI, adverse events such as renal dysfunction, thrombotic and/or bleeding events, and thrombocytopenia can be common due to the use of various types of drugs. In this case, adverse events made TTP diagnosis difficult, even though there was a short gap between index manifestations and subsequent features. After consulting with nephrology and hematology, the presumptive diagnosis of TTP was confirmed based on cardinal manifestations, including fever, disturbed mental status, oliguria, renal dysfunction, anemia, and thrombocytopenia.

TTP-induced cardiac morbidity has a high mortality rate, and diagnosing and managing the condition early is crucial. Research has shown that TTP patients with high levels of positive cardiac biomarkers have a higher risk of severe morbidity and mortality. Troponin levels greater than 0.25 ng/mL have been linked to a threefold increase in mortality risk for TTP patients [9,10]. Managing myocardial infarction in TTP patients can be challenging due to low platelet count, which makes initiating dual antiplatelet therapy, including aspirin and clopidogrel, more complex [2]. Antiplatelet medicine further increases the bleeding risk in TTP patients. Although adding aspirin to dual antiplatelet therapy has been found to inhibit platelet aggregation and decrease the death rate in TTP patients, adding clopidogrel to the treatment regimen is contentious since it can cause TTP itself. Recent research suggests that adding ticlopidine to plasmapheresis can reduce mortality at day 15 [11]. Aspirin can be given as an adjuvant therapy when the platelet count is above 50,000/µL, with no risk of bleeding. Managing myocardial infarction in TTP patients can be complicated by severe thrombocytopenia and acute kidney failure, which can impede dual antiplatelet therapy, cardiac catheterization, or percutaneous coronary intervention [12]. Standard recommendations suggest conducting a complete cardiac workup with clinical examination, EKG, echocardiography, and serum assessment of cardiac enzymes [13]. In TTP patients with myocardial injury, immediate plasmapheresis is necessary to prevent further cardiac injury and mortality. Additionally, continuous cardiac monitoring is essential in these patients, given the increased risk of fatal arrhythmias [14].

## Conclusions

NSTEMI, as an initial manifestation of TTP, is a potentially serious medical condition. Despite the rarity, TTP complications must be considered among other possible causes of unexpected thrombocytopenia during acute phase treatment of acute coronary syndrome because of high morbidity and mortality. Early initiation of plasmapheresis and steroid administration should be instituted promptly, and all the patients require careful evaluation and monitoring of EKG, cardiac enzymes, telemetry, and coagulation data.
